# Combined use of Donepezil and Memantine increases the probability of five-year survival of Alzheimer’s disease patients

**DOI:** 10.1038/s43856-024-00527-6

**Published:** 2024-05-23

**Authors:** Ehsan Yaghmaei, Hongxia Lu, Louis Ehwerhemuepha, Jianwei Zheng, Sidy Danioko, Ahmad Rezaie, Seyed Ahmad Sajjadi, Cyril Rakovski

**Affiliations:** 1 Schmid College of Science and Technology, Chapman University, Orange, CA USA; 2 School of Medicine, University of North Carolina at Chapel Hill, Chapel Hill, NC USA; 3 Children’s Hospital of Orange County (CHOC), Orange, CA USA; 4 School of Medicine, University of California, Irvine, CA USA

**Keywords:** Alzheimer's disease, Alzheimer's disease

## Abstract

**Background:**

Alzheimer’s disease (AD) is the most common neurodegenerative disease. Studying the effects of drug treatments on multiple health outcomes related to AD could be beneficial in demonstrating which drugs reduce the disease burden and increase survival.

**Methods:**

We conducted a comprehensive causal inference study implementing doubly robust estimators and using one of the largest high-quality medical databases, the Oracle Electronic Health Records (EHR) Real-World Data. Our work was focused on the estimation of the effects of the two common Alzheimer’s disease drugs, Donepezil and Memantine, and their combined use on the five-year survival since initial diagnosis of AD patients. Also, we formally tested for the presence of interaction between these drugs.

**Results:**

Here, we show that the combined use of Donepezil and Memantine significantly elevates the probability of five-year survival. In particular, their combined use increases the probability of five-year survival by 0.050 (0.021, 0.078) (6.4%), 0.049 (0.012, 0.085), (6.3%), 0.065 (0.035, 0.095) (8.3%) compared to no drug treatment, the Memantine monotherapy, and the Donepezil monotherapy respectively. We also identify a significant beneficial additive drug-drug interaction effect between Donepezil and Memantine of 0.064 (0.030, 0.098).

**Conclusions:**

Based on our findings, adopting combined treatment of Memantine and Donepezil could extend the lives of approximately 303,000 people with AD living in the USA to be beyond five-years from diagnosis. If these patients instead have no drug treatment, Memantine monotherapy or Donepezil monotherapy they would be expected to die within five years.

## Plain language summary

Alzheimer’s disease is the most common type of dementia, affecting millions of people worldwide. In this study, we investigated the effects of two drugs commonly prescribed to people with Alzheimer’s disease called Donepezil and Memantine to see whether they had an impact on when people died. We found that the combined use of Donepezil and Memantine significantly increased the probability of a person surviving five years compared to no drug treatment or treatment with Donepezil or Memantine alone. Our results suggest that the lives of many Alzheimer’s patients in the USA who are currently on no drug treatment or just Donepezil or Memantine could be extended if they were treated with both drugs simultaneously.

## Abstract (web summary)

Yaghmaei et al. utilize doubly robust causal inference estimators and a large medical data set to evaluate the impact of combining Donepezil and Memantine on five-year survival since initial diagnosis of Alzheimer’s disease patients. A significant benefit to survival is seen when the drugs are combined.

## Introduction

Alzheimer’s disease is the most prevalent type of dementia that affects over 50 million individuals globally. AD is among the five leading causes of death worldwide and the sixth (seventh during the COVID-19 pandemic) in the United States^1,2^. The Alzheimer’s Association reported that in the year 2022, AD affected more than 6.5 million individuals in the United States^3^. It has been projected that this figure will reach 14 million by the year 2060^4^.

Alzheimer’s disease research has been multifaceted with focal points on identification of causes, genetic and environmental risk factors, diagnostic methods, and treatment^5–10^. Despite major advances in our understanding of genetic and environmental risk factors, pathological features, and diagnostic methods, the currently available treatments are largely limited to alleviating AD symptoms. In addition to recently approved anti amyloid antibody lecanemab, Choline esterase inhibitors (Donepezil, Rivastigmine, and Galantamine) and anti *N*-methyl-d-aspartate (NMDA) receptor antagonists (memantine) are the only two classes of Food and Drug Administration (FDA) approved Alzheimer’s disease pharmacological treatments^11–13^. Choline esterase inhibitors (ChEI) increase the availability of acetylcholine in synaptic space and are generally considered cognitive enhancers. There have been multiple studies that have shown evidence that these drugs reduce mortality in AD patients and discuss the possible mechanisms of achieving that beneficial effect^14–16^. Memantine, on the other hand, regulates the activity of glutamate, an excitatory neurotransmitter, and is mainly used to treat agitation and sundowning that is common is more advanced stages of AD. Previous studies have suggested that Donepezil and Memantine might have complementary mechanisms of action in treating Alzheimer’s disease^17–19^. Furthermore, a recent causal inference study assessing the effect of the combined treatment of Donepezil and Memantine on hospital and emergency department visits of Alzheimer’s disease patients has shown that the use of Donepezil and Memantine treatment significantly reduces the average number of hospital or emergency department visits per year compared to no drug treatment, Memantine monotherapy and Donepezil monotherapy respectively^20^.

In a recent meta-analysis study, the combined use of Donepezil and Memantine in Alzheimer’s disease patients has been shown to significantly improve cognition^21^. Another related study also investigated the use of Donepezil and Memantine in Alzheimer’s disease patients and found that the combined treatment improved cognitive function, reduced the need for caregiver assistance and delayed nursing home placement^22^. Other endeavors have investigated the efficacy and safety of these drugs^23,24^. However, as far as we are aware, no previous research has addressed the causal effect of the combined use of Donepezil and Memantine on AD patient survival.

In this paper, we describe a comprehensive causal inference study in which we apply doubly robust estimators combined with nonparametric bootstrapping confidence intervals to a large medical database to investigate the causal effect of the combined use of Donepezil and Memantine on AD patient survival. The causal inference framework applied to observational data attains bias-free estimates of treatment effects by identifying the sources of bias, explicitly modeling the causal relationships among variables, and removing the bias via multi-stage statistical methods. The successful use of this framework is predicated on specifying and implementing a correct statistical model for a particular outcome variable of interest such as survival odds or probability of receiving a particular treatment. We implement the doubly-robust causal inference approach in the study that combines estimates from two distinct causal inference methods that use two different statistical models and yields an unbiased estimate of treatment even if only one of these statistical models is correctly specified^25^. Using this method, we show that the combined use of Donepezil and Memantine significantly elevates the probability of five-year survival compared to no drug treatment, Memantine monotherapy, and Donepezil monotherapy respectively. We also identify a significant beneficial additive drug-drug interaction effect between Donepezil and Memantine.

## Methods

### Data

Our data were queried from the Oracle EHR Real-World Data, one of the largest, multi-center, high quality research medical databases. The Oracle EHR Real-World Data is a secondary dataset designed, curated, and maintained as a de-identified, HIPAA compliant research dataset. Oracle maintains RWD governing policies with healthcare providers via data network and data use agreements to clearly define data rights and restrictions with specific regard to HIPAA privacy and security standards and safeguards. All research studies performed using the Oracle EHR Real-World Data are therefore considered to be exempt from IRB review. It has already provided the data foundation for numerous analytical breakthroughs^26–28^.

### Oracle EHR Real-World Data

Oracle EHR Real-World Data is a large collection of de-identified electronic health records obtained from over 110 health systems in the United States. As of May 2022, the Oracle EHR is comprised of over 100 million patients and 1.5 billion encounters^27,28^. The database contains conditions, medications, procedures, and lab tables with more than 2.3 billion, 2.9 billion, 486 million, and 11.5 billion records, respectively. The size and longitudinal structure of the Oracle EHR data provide an ideal platform for applications of advanced statistical methods and artificial intelligence approaches in medical research.

### Alzheimer’s disease dataset

**Fig. 1:**
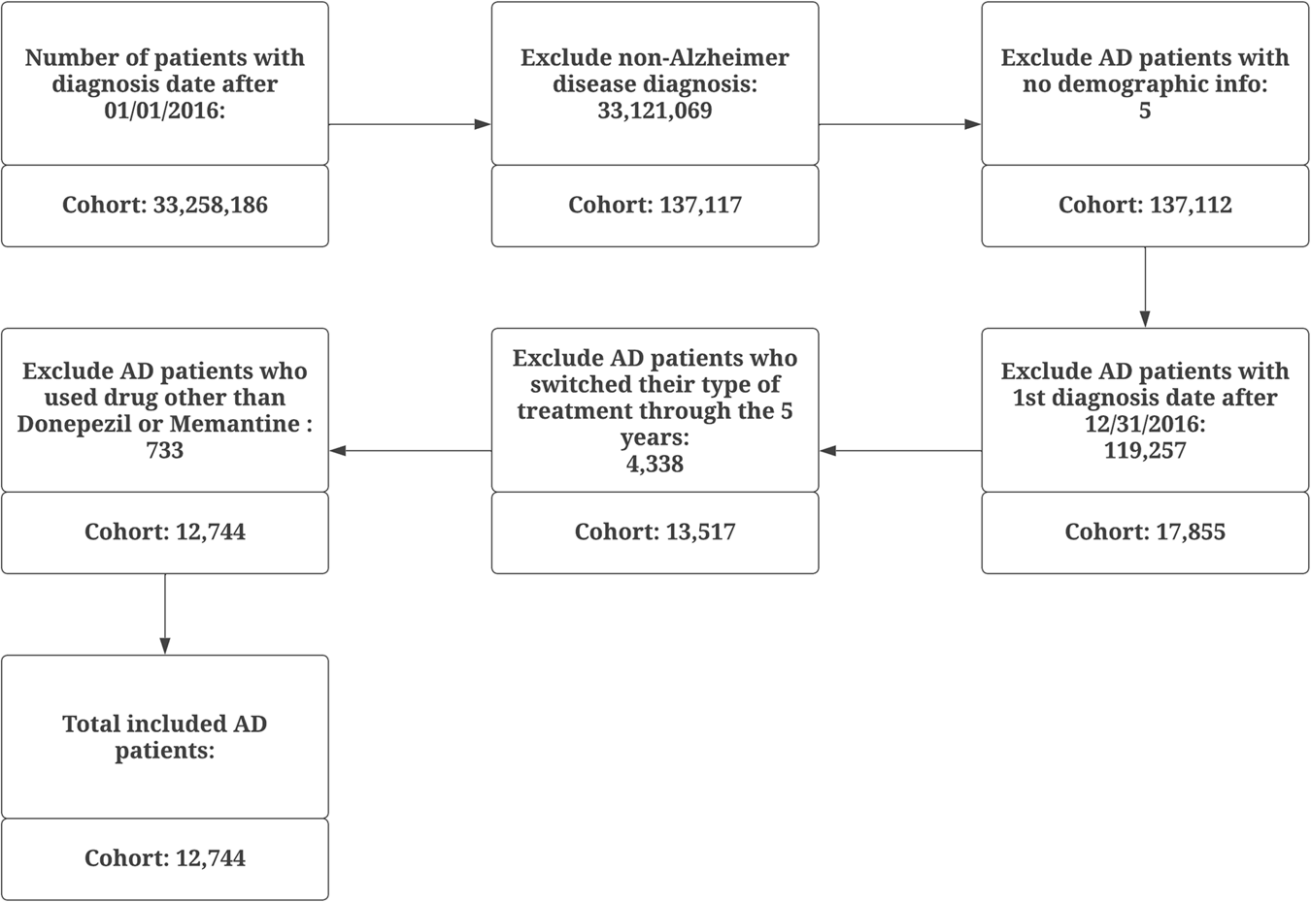
Flowchart of data preprocessing steps. The boxes describe all the filtering steps in the data preprocessing stage. The bottom part of each box reflects the current sample size after implementing the filtering step described in the top part of the same box. The arrows connect adjacent steps of the data preprocessing. The last box shows the size of the final cohort after applying the filters.

Firstly, we extracted information on patients with Alzheimer’s disease (using International Classification of Diseases (ICD)−10 Code G30 and ICD-9 code 331) with a first (new) diagnosis date after 1 January 2016 which yielded a total of 137,117 patient IDs. Patients with missing demographic information and subjects with first diagnosis that occurred after 12/31/2016 were subsequently excluded. These steps produced a cohort of 17,855 study subjects with a follow-up time of at least five years. Next, 4338 (24.3%) patients who switched treatments throughout the duration of the study period were removed which resulted in a cohort of size 13,517. This study was focused on patients receiving the two main drugs, Donepezil and Memantine, their combination, and a control group consisting of patients who received no drug treatment. The final cohort size after applying all filters was 12,744 patients. A flowchart describing the stepwise data preprocessing process is shown in Fig. 1.

### Outcome variable (5-year survival after initial diagnosis)

We used data on 12,774 patients with confirmed first diagnosis of Alzheimer’s disease in 2016. That ensured the necessary five-year follow-up time duration of the subjects as the database only included data up to 2022. Among these patients, 9940 (78.00%) survived five years after their initial AD diagnosis. The outcome variable of interest was the indicator of the five-year survival of the study participants.

### Alzheimer’s disease medications

We only considered the most prevalent five Alzheimer’s disease medications: Donepezil and Memantine, Galantamine, Memantine, Rivastigmine, and Donepezil. Approximately half of the patients (45.54%) received no drug treatment. The most commonly prescribed Alzheimer’s disease drug was Donepezil with 28.94%, followed by Memantine with 10.7% and combined use of Donepezil and Memantine with 9.11%. Detailed summary statistics of the patient AD medications use are shown in Table 1.

**Table 1:** Summary statistics of the AD patient medications use

Medications	*N* (%)^a^
None	6155 (45.54)
Donepezil	3912 (28.94)
Memantine	1446 (10.7)
Donepezil & Memantine	1231 (9.11)
Rivastigmine	364 (2.69)
Memantine & Rivastigmine	170 (1.26)
Galantamine	114 (0.84)
Memantine & Galantamine	47 (0.35)
Donepezil -Memantine Single Med	42 (0.31)
Donepezil & Rivastigmine	10 (0.07)
Donepezil & Donepezil-Memantine Single Med	8 (0.06)
Donepezil & Memantine & Rivastigmine	6 (0.04)
Donepezil & Memantine & Donepezil-Memantine Single Med	6 (0.04)
Memantine & Donepezil-Memantine Single Med	2 (0.01)
Donepezil & Galantamine	2 (0.01)
Donepezil & Memantine & Rivastigmine & Galantamine	1 (0.01)
Donepezil & Memantine & Galantamine	1 (0.01)
Total	13,517 (100)

^a^*N* (%) represent the frequency count of each medication use and the corresponding percent of each medication use in the total sample.

We included a list of expertly selected comorbidities that are potential confounders for the effect of Alzheimer’s disease treatment of the five-year survival of patients. Comorbidities were collected at the time of their occurrence which could have happened before or after the initial AD diagnosis. The most prevalent comorbidity was acute kidney injury and chronic kidney disease accounting for 27.45% of the patients, followed by heart disease with 23.58% and cerebral infarction with 14.51%. Detailed summary statistics of the AD patient comorbidities are shown in Table 2.

**Table 2:** Summary statistics of the AD patient comorbidities

	No drug treatment	Memantine	Donepezil	Donepezil/Memantine	Total
Comorbidities^a^	*N* (%)^b^	*N* (%)^b^	*N* (%)^b^	*N* (%)^b^	*N* (%)^b^
Cerebral infarction (I60–I69)					
Absence	5276 (85.72)	1228 (84.92)	3335 (85.25)	1064 (86.43)	10,903 (85.55)
Presence	879 (14.28)	218 (15.08)	577 (14.75)	167 (13.57)	1841 (14.45)
Diabetes (E10–E13)					
Absence	5453 (88.59)	1286 (88.93)	3432 (87.73)	1096 (89.03)	11,267 (88.41)
Presence	702 (11.41)	160 (11.07)	480 (12.27)	135 (10.97)	1477 (11.59)
Overweight and obesity (E66)					
Absence	6064 (98.52)	1419 (98.13)	3839 (98.13)	1209 (98.21)	12,531 (98.33)
Presence	91 (1.48)	27 (1.87)	73 (1.87)	22 (1.79)	213 (1.67)
Hypertensive diseases (I10)					
Absence	5425 (88.14)	1264 (87.41)	3372 (86.2)	1061 (86.19)	11,122 (87.27)
Presence	730 (11.86)	182 (12.59)	540 (13.8)	170 (13.81)	1622 (12.73)
Other forms of heart disease (I3–I5)					
Absence	4734 (76.91)	1085 (75.03)	2939 (75.13)	964 (78.31)	9722 (76.29)
Presence	1421 (23.09)	361 (24.97)	973 (24.87)	267 (21.69)	3022 (23.71)
Acute kidney injury and chronic kidney disease (N17–N19)					
Absence	4488 (72.92)	1062 (73.44)	2790 (71.32)	897 (72.87)	9237 (72.48)
Presence	1667 (27.08)	384 (26.56)	1122 (28.68)	334 (27.13)	3507 (27.52)
Total	6155 (100)	1446 (100)	3912 (100)	1231 (100)	12,744 (100)

^a^Designations in parenthesis are ICD-10 codes.

^b^*N* (%) represent the frequency count of each comorbidity and the corresponding percent of each comorbidity within treatment and in the total sample.

### Demographic characteristics

Alzheimer’s disease was ascertained using the following ICD-10 codes: G30 (Alzheimer’s disease), G30.0 (Alzheimer’s disease with early onset), G30.1 (Alzheimer’s disease with late onset), G30.8 (Other Alzheimer’s disease), and G30.9 (Alzheimer’s disease, unspecified) from January 2016 to December 2022 for a total of 12,774 patients. Demographics characteristics were collected at the time of the first AD diagnosis. The distribution of patients across age groups was as follows: 10,264 (80.54%) were aged 76-85 years, 1834 (14.39%) were aged 66–75 years, 607 (4.76%) were aged 46–65 years, and 39 (0.31%) were aged 45 years or younger. The patient population included 7967 (62.52%) females, 4772 (37.45%) males, and for the remaining 5 (0.04%) the sex was unknown. The racial distribution of the study population was as follows: 10,341 (81.14%) Caucasian, 808 (6.34%) African American, 279 (2.19%) Asian, 44 (0.35%) Hispanic, 42 (0.33%) American Indian or Alaska Native, 20 (0.16%) Pacific Islander, and 1210 (9.49%) were Unknown. Detailed summary statistics of the AD patient demographic characteristics are shown in Table 3.

**Table 3:** Summary statistics of the AD patient demographic characteristics

	No drug treatment	Memantine	Donepezil	Donepezil/Memantine	Total
	*N* (%)^a^	*N* (%)^a^	*N* (%)^a^	*N* (%)^a^	*N* (%)^a^
Age at diagnosis					
<46	32 (0.52)	1 (0.07)	6 (0.15)	(0)	39 (0.31)
46–65	291 (4.73)	57 (3.94)	206 (5.27)	53 (4.31)	607 (4.76)
66–75	792 (12.87)	212 (14.66)	630 (16.1)	200 (16.25)	1834 (14.39)
76–85	5040 (81.88)	1176 (81.33)	3070 (78.48)	978 (79.45)	10,264 (80.54)
Gender					
Female	3833 (62.27)	932 (64.45)	2479 (63.37)	723 (58.73)	7967 (62.52)
Male	2319 (37.68)	514 (35.55)	1431 (36.58)	508 (41.27)	4772 (37.45)
Unknown	3 (0.05)	(0)	2 (0.05)	(0)	5 (0.04)
Race					
African American	400 (6.5)	74 (5.12)	273 (6.98)	61 (4.96)	808 (6.34)
American Indian or Alaska Native	19 (0.31)	6 (0.41)	11 (0.28)	6 (0.49)	42 (0.33)
Asian	138 (2.24)	35 (2.42)	74 (1.89)	32 (2.6)	279 (2.19)
Caucasian	4945 (80.34)	1168 (80.77)	3200 (81.8)	1028 (83.51)	10,341 (81.14)
Hispanic	23 (0.37)	5 (0.35)	13 (0.33)	3 (0.24)	44 (0.35)
Pacific Islander	10 (0.16)	(0)	9 (0.23)	1 (0.08)	20 (0.16)
Unknown	620 (10.07)	158 (10.93)	332 (8.49)	100 (8.12)	1210 (9.49)
Marital status					
Divorced	442 (7.18)	73 (5.05)	286 (7.31)	66 (5.36)	867 (6.8)
Married	2002 (32.53)	538 (37.21)	1463 (37.4)	528 (42.89)	4531 (35.55)
Single	3185 (51.75)	684 (47.3)	1867 (47.72)	522 (42.4)	6258 (49.11)
Unknown	526 (8.55)	151 (10.44)	296 (7.57)	115 (9.34)	1088 (8.54)
Total	6155 (100)	1446 (100)	3912 (100)	1231 (100)	12,744 (100)

^a^*N* (%) represent the frequency count of each demographic characteristic and the corresponding percent of each demographic characteristic within treatment and in the total sample.

### Treatment stratified mortality rates per 1000 patient-years

We calculated the unadjusted mortality rates per treatment group per 1000 person-years. The lowest mortality rate of 41.47 was attained in the Donepezil-Memantine treatment group. The mortality rates for patients on no drug treatment, Memantine and Donepezil monotherapies were 36%, 32%, and 42% higher, respectively. Details are shown in Table 4.

**Table 4:** Mortality rates per 1000 patient-years

Medications	Mortality rate per 1000 patient-years
No drug treatment	55.96
Memantine	54.80
Donepezil	58.66
Donepezil-Memantine	41.47
Total	55.21

### Causal inference model

The institutional review board of Children’s Health of Orange County (institutional review board no. 2109133) approved this retrospective cohort study and waived the informed consent requirement because the database used was deidentified and the study was not classified as human subject research and posed no more than minimal risk to patients.

Causal inference aims at bias-free estimation of the effects of interventions or treatments on an outcome of interest. It removes bias by addressing the two main issues that affect observational studies, confounding and selection bias^29^. In this work, we implemented the doubly robust estimator method to assess the causal effects of Donepezil, Memantine and their combined use on the five-year survival of AD patients. This approach provides an advantageous alternative to single-model approaches as it combines distinct estimates (obtained via inverse probability weighting and standardization) into a doubly robust estimate that is unbiased even if only one of the two underlying statistical models used in inverse probability weighting and standardization is correctly specified^25^. A detailed description of the causal effect estimation, a multiple treatment comparison as well as the computations of the corresponding naïve and Bonferroni-adjusted 95% confidence intervals (using nonparametric bootstrapping) are presented in the subsequent sections. Lastly, we derive risk difference estimates and additive drug-drug interaction estimates together with their corresponding 95% naïve and Bonferroni-adjusted confidence intervals.

### Treatments

We only considered treatments used by at least 0.5% of the patients and we removed patients that switched treatments at any point of the follow-up period. As we removed the treatment switchers from the study, the remaining treatments are not time varying. The rest of the covariates are not considered time varying. This study design has been adopted by the original causal inference study on the effect of smoking cessation on weight loss after 10 years^25^. The follow-up period of this study is twice as long as the follow-up period of ours. Therefore, the considered treatments 0, 1, 2, and 3 were defined as follows: no drug, Memantine monotherapy, Donepezil monotherapy, and combined use of Memantine and Donepezil, respectively. Specifically, 6155 patients (48.30%) were on treatment 0, 3912 (30.70%) were on treatment 1, 1446 (11.35%) were on treatment 2, and 1231 (9.66%) were on treatment 3.

### Statistics and reproducibility

Let $Y$ denote the five-year survival status of a patient and $a=0,1,2,3$ denote the treatments. We implement doubly robust estimator to assess the mean counterfactual outcomes under all treatments,

1.1 $$E({Y}^{a=i}),i=0,1,2,3$$

This approach combined estimates from two singly robust causal inference estimators, Inverse Probability (IP) weighting and standardization. Let $L$ denote a vector of confounders that encompasses demographic variables, comorbidities, and the squares of the continuous variables. First, we describe the inverse probability (IP) weighted estimators of the mean effects of all treatments. We utilize the following multinomial logistic regression model,

1.2 $$\log [P(A=i|L)/P(A=0|L)]={\beta }_{i}^{T}L,i=1,2,3$$

We use this model to estimate the probabilities of receiving the three drug treatments,

1.3 $$\hat{P}(A=i|L)=\frac{{e}^{{\hat{\beta }}_{i}^{T}L}}{1+{\sum }_{j=1}^{3}{e}^{{\hat{\beta }}_{j}^{T}L}}={\hat{\pi }}_{i}(L),i=1,2,3$$

as well as the probability of receiving no drug treatment,

1.4 $$\hat{P}(A=0|L)=\frac{1}{1+{\sum }_{j=1}^{3}{e}^{{\hat{\beta }}_{j}^{T}L}}={\hat{\pi }}_{0}(L)$$

The Inverse Probability (IP) weights are defined as ${[{\pi }_{i}(L)]}^{-1}$. Then, corresponding IP weighted means can be written as:

1.5 $$E({Y}^{a=i})=E\left(\frac{{A}_{i}Y}{{\pi }_{i}(L)}\right)$$

Further, we estimate these IP weighted means via the Horwitz-Thompson IP weighted estimators:

1.6 $${\hat{E}}_{IP}({Y}^{a=i})=\hat{E}\left(\frac{{A}_{i}Y}{{\pi }_{i}(L)}\right)=\frac{1}{n}{\sum }_{k=1}^{n}\frac{I\{{A}_{k}=i\}{Y}_{k}}{{\hat{\pi }}_{i}(L)}$$

Next, we describe the standardized estimators of the mean effects of all treatments. Let ${b}_{i}(L)$ denote the mean value of $Y$ for patients in stratum $L$ that are on treatment $i$,

1.7 $${b}_{i}(L)=E(Y|A=i,L)$$

We can estimate these values via the following standard logistic regression model,

1.8 $$\log [P(Y=1|A,L)/P(Y=0|A,L)]={\alpha }_{1}^{T}L+{\alpha }_{2}^{T}A+{\alpha }_{3}^{T}AL$$

1.9 $${\hat{b}}_{i}(L)=\frac{{e}^{{\hat{\alpha }}_{1}^{T}L+{\hat{\alpha }}_{2}^{T}A+{\hat{\alpha }}_{3}^{T}AL}}{1+{e}^{{\hat{\alpha }}_{1}^{T}L+{\hat{\alpha }}_{2}^{T}A+{\hat{\alpha }}_{3}^{T}AL}}$$

Using the double expectation formula, $E({Y}^{a=i})$ can be written as,

1.10 $$E({Y}^{a=i})=E[E(Y|A=i,L)]=E[{b}_{i}(L)]$$

We can calculate the standardized estimators of these means using the plug-in g-formula:

1.11 $${\hat{E}}_{ST}({Y}^{a=i})=\frac{1}{n}{\sum }_{k=1}^{n}{\hat{b}}_{i}(L)$$

Finally, the Doubly Robust Estimator of $E({Y}^{a=i})$ is given by,

1.12 $${\hat{E}}_{DR}({Y}^{a=i})=\frac{1}{n}{\sum }_{k=1}^{n}[{\hat{b}}_{i}({L}_{k})+\frac{I\{{A}_{k}=i\}}{{\hat{\pi }}_{i}(L)}({Y}_{k}-{\hat{b}}_{i}({L}_{k}))]$$

Thus, we can subsequently estimate the three causal risk differences that contrast treatment,

1.13 $$R{D}_{s}=E({Y}^{a=3})-E({Y}^{a=s}),s=0,1,2$$

1.14 $$\hat{R}{D}_{s}={\hat{E}}_{DR}({Y}^{a=3})-{\hat{E}}_{DR}({Y}^{a=s})$$

### Nonparametric bootstrap 95% confidence intervals for the average counterfactuals and causal effects

We calculated 95% confidence intervals for the counterfactual outcomes under all treatments using nonparametric bootstrapping. In particular, we obtained 10,000 datasets *B*_1_, *B*_2_,...,*B*_10,000_ of size 12,744 by implementing random sampling of patients with replacement from the original data. We applied the Doubly Robust estimator (1.12) to all *B*_i_’s, *i* = 0, 1,2,3 to obtain 10,000 estimates of the counterfactual outcome of the $i$ th treatment,

1.15 $${C}_{ij}={\hat{E}}_{DR}^{{B}_{j}}({Y}^{a=i}),j=1,2,{{{{\mathrm{..}}}}}.,10,000$$

These values empirically estimate the distribution of the mean counterfactual outcome under treatment $i$, ${\hat{E}}_{DR}({Y}^{a=i})$. Furthermore, the empirical 95% confidence interval for the mean counterfactual outcome for the $i$ th treatment is given by:

1.16 $$({C}_{0.025}^{i},{C}_{0.975}^{i})$$

where ${C}_{0.025}^{i}$ and ${C}_{0.975}^{i}$ are the 2.5th and the 97.5th percentiles of the samples defined in (1.15).

Them, the Bonferroni adjusted (for four treatments) simultaneous 95% confidence intervals for the mean counterfactual outcomes are given by,

1.17 $$({C}_{0.00625}^{i},{C}_{0.99375}^{i}),i=0,1,2,3$$

Similarly, we obtain empirical 95% confidence intervals and the Bonferroni adjusted (for three treatment differences) 95% confidence intervals for the differences of the mean counterfactual outcomes between treatment 3 and treatment $s$, $s=0,1,2$.

### Drug-drug interaction analysis

We assess the presence of drug-drug interaction (DDI) by evaluating the following expression,

1.18 $$DDI=E({Y}^{a=3})-E({Y}^{a=2})-E({Y}^{a=1})+E({Y}^{a=0})$$

We use the doubly robust estimates (1.12) for each counterfactual outcome in (1.18),

1.19 $$D\hat{D}I={\hat{E}}_{DR}({Y}^{a=3})-{\hat{E}}_{DR}({Y}^{a=2})-{\hat{E}}_{DR}({Y}^{a=1})+{\hat{E}}_{DR}({Y}^{a=0})$$

Finally, we derive the 95% confidence interval for the $DDI$ measure by implementing the same nonparametric bootstrapping procedure described above.

### Sensitivity analysis

We assess the robustness of the reported associations to potential unmeasured and uncontrolled confounding. We use the E-Value measure which is the defined as the minimum strength of association on the risk ratio scale that a missing confounder would need to have with both the treatment and outcome variables to completely explain away the treatment outcome association^30^. In particular, for a calculated risk ratio $$R{\hat{R}}_{i}=\frac{\hat{P}({Y}^{a=3}=1)}{\hat{P}({Y}^{a=i}=1)}\,$$ and corresponding 95% Bonferroni adjusted confidence interval $$(L{L}_{i},L{R}_{i})$$$$,\,i=0,1,2$$, the corresponding E-Values for the estimate and confidence interval are given by:

1.20 $$E-Valu{e}_{i}=R{\hat{R}}_{i}+\sqrt{R{\hat{R}}_{i}\,(1-R{\hat{R}}_{i})}$$

1.21 $$E-Valu{e}_{C{I}_{i}}=L{L}_{i}+\sqrt{L{L}_{i}\,(1-L{L}_{i})}$$

### Reporting summary

Further information on research design is available in the Nature Portfolio Reporting Summary linked to this article.

## Results

The IP weighing step achieves almost perfectly even distribution of all 10 confounders (Gender, age at diagnosis, race, marital status, overweight and obesity, hypertensive diseases, acute kidney injury and chronic kidney disease, diabetes, cerebral infarction, and other forms of heart disease) across all four treatment groups. The corresponding 60 standardized mean differences have a mean of 0.001, standard deviation of 0.027, a minimum of −0.10, and a maximum of 0.09. Details are shown in Table 5.

**Table 5:** Covariates distribution comparison across all treatments in the pseudo population using standardized mean differences

Covariates	No drug vs. Memantine	Memantine vs. Donepezil	Donepezil vs. Memantine/Donepezil	No drug vs. Donepezil	No drug vs. Memantine/Donepezil	Memantine vs. Memantine/Donepezil
Gender	0	−0.01	0.02	−0.01	0.01	0.01
Age at diagnosis	−0.03	0.01	0.06	−0.02	0.05	0.08
Race	0.1	−0.1	0	0	−0.01	−0.1
Marital status	0	0	0	0	0	0
Overweight and obesity (E66)^a^	0	0	−0.01	0	−0.01	−0.01
Hypertensive diseases (I10)^a^	0	−0.01	0	0	0	0
Acute kidney failure (N17–N19)^a^	−0.01	0.01	0	0	0	0.01
Diabetes (E10–E13)^a^	0	0	0.01	0	0.01	0.01
Cerebral infarction (I60–I69)^a^	0	0	0	0	0	0
Other forms of heart disease (I3–I5)^a^	0	0	0	0	0	0

^a^Designations in parenthesis are ICD-10 codes.

Furthermore, the IP weighted and standardized estimators yield extremely close counterfactual estimates for all treatments (0.780, 0.779), (0.779, 0.781), (0.767, 0.768), and (0.831, 0.827), thus providing an indirect support for the correct specification of the two different statistical models that these two approaches implement.

Our results from the doubly robust treatment analyses show that the estimated counterfactual probabilities for five-year survival (and Bonferroni adjusted 95% CI) under no drug use (treatment 0), single drug use of Memantine (treatment 1), single drug use of Donepezil (treatment 2), and combined use of Donepezil and Memantine (treatment 3) were 0.780 (0.767, 0.793), 0.781 (0.754, 0.808), 0.765 (0.748, 0.782) and 0.830 (0.803, 0.856) respectively. Potential explanations for lack of difference between memantine alone and no drug treatment groups are that those only on memantine are either at the more advanced stages of the disease or have contra indications to Aricept such as having heart block. Both explanations can affect life expectancy. In terms of donepezil alone group, there is a possibility that these individuals had died at the earlier stages of their Alzheimer’s disease due to alternative reasons. Details from the analyses of the average counterfactual outcomes under all treatments are shown in Table 6.

**Table 6:** Average counterfactual probabilities of five-year survival under all treatments and 95% confidence intervals (*n* = 12,744)

Average counterfactuals^a^	Estimates	95% confidence intervals	Bonferroni adjusted 95% confidence intervals
$E({Y}^{a=0})$	0.780	(0.769, 0.790)	(0.767, 0.793)
$E({Y}^{a=1})$	0.781	(0.760, 0.802)	(0.754, 0.808)
$E({Y}^{a=2})$	0.765	(0.751, 0.778)	(0.748, 0.782)
$E({Y}^{a=3})$	0.830	(0.808, 0.851)	(0.803, 0.856)

^a^$E({Y}^{a=i})$ is the average counterfactual value for treatment *i*, where *i* = 0, 1, 2, 3, where 0 = No drug treatment, 1 = Memantine, 2 = Donepezil, 3 = Memantine and Donepezil.

Our results from the treatment comparison analyses show that the combined use of Donepezil and Memantine significantly increased the probability of five-year survival of Alzheimer’s disease patients compared to no drug use and single drug use of Memantine and Donepezil. In particular, compared to no drug use, the combined use of Memantine and Donepezil significantly increased the probability of five-year survival by 0.05 (6.4%) with corresponding Bonferroni adjusted 95% CI (0.021, 0.078). Further, compared to use of Memantine alone, the combined use of Memantine and Donepezil significantly increased the probability of five-year survival by 0.049 (6.3%) with corresponding Bonferroni adjusted 95% CI (0.012, 0.085). Lastly, compared to use of Donepezil alone, the combined use of Memantine and Donepezil significantly increased the probability of five-year survival by 0.065 (8.3%) with corresponding Bonferroni adjusted 95% CI (0.035, 0.095). Details from the analyses of the differences of causal effects are shown in Table 7.

**Table 7:** Causal effects and 95% confidence intervals (*n* = 12,744)

Causal effects^a^	Estimates	95% Confidence intervals	Bonferroni adjusted 95% confidence intervals
$E({Y}^{a=3})-E({Y}^{a=0})$	0.050	(0.026, 0.073)	(0.021, 0.078)
$E({Y}^{a=3})-E({Y}^{a=1})$	0.049	(0.019, 0.078)	(0.012, 0.085)
$E({Y}^{a=3})-E({Y}^{a=2})$	0.065	(0.040, 0.089)	(0.035, 0.095)

^a^$E({Y}^{a=i})$ is the average counterfactual value for treatment *i*, where *i* = 0, 1, 2, 3. where 0 = No drug treatment, 1 = Memantine, 2 = Donepezil, 3 = Memantine and Donepezil.

Our results from the drug-drug interaction analysis shows that Memantine and Donepezil have significant beneficial interaction with respect to the five-year survival of Alzheimer’s disease patients. The estimated value of the interaction effect calculated using (1.18) was 0.064 with 95% confidence interval (0.030, 0.098).

### Sensitivity analysis results

The calculated risk ratios of 1.064, 1.063, and1.085 and corresponding Bonferroni adjusted 95% confidence intervals (1.026, 1.100), (1.016, 1.112) and (1.045, 1.126) show the significant beneficial effect of treatment 3 compared to the treatment 0, 1 and 2 on the risk ratio scale. Moreover, the corresponding E-values for the risk ratios and corresponding confidence intervals were 1.325, 1.321, 1.389, 1.191, 1.142, and 1.261 respectively. Details are presented in Table 8. Therefore, the estimated causal effect could be explained away by an unmeasured confounder that is associated with treatment and outcome through risk ratios of 1.389 each. The confidence intervals could be moved to include the null value of one by an unmeasured confounder that was associated with both treatment and outcome through risk ratios of 1.261 each.

**Table 8:** E-Value calculations based on casual risk ratios and confidence interval estimates (*n* = 12,744)

Risk ratios	Bonferroni adjusted 95% confidence intervals	*E*-value for ratio	*E*-value for CI
1.064	(1.026, 1.100)	1.325	1.191
1.063	(1.016, 1.112)	1.321	1.142
1.085	(1.045, 1.126)	1.389	1.261

## Discussion

This study used a doubly robust causal inference approach to investigate the causal effect of the combined use of Donepezil and Memantine on the survival of Alzheimer’s disease patients. The results of our study demonstrated that the combined use of these two drugs significantly increased the probability of five-year survival of Alzheimer’s disease patients, suggesting the potential benefits of using combination therapy for the treatment of Alzheimer’s disease.

As reported by the Alzheimer’s disease Association, there are an estimated 6.5 million patients with Alzheimer’s disease in the United States^3^. The adoption of the combined treatment of Memantine and Donepezil in the U.S. could extend the lives of approximately 303,000 current AD patients above the five-year threshold since diagnosis that would have otherwise expired. Specifically, the combined treatment could extend the lives of approximately 157,000, 98,000, and 48,000 current AD patients that are currently on no drug treatment, Memantine monotherapy and Donepezil monotherapy, respectively. The Centers for Disease Control and Prevention (CDC) projected number of Alzheimer’s disease patients in 2060 is 14 million in the U.S, and the estimated number of lives extended will be ~652,000.

However, it is worth noting that our study has some potential limitations with respect to both confounding and selection bias. Firstly, the study relied on electronic health records and did not include information on lifestyle factors, genetic, and environmental exposure variables that could be common causes of treatment and survival. Similarly, the database did not include an AD severity measurement. Further, the follow-up period spanned pre-COVID and COVID time periods. This time variable could potentially modify the effects of the four treatments. Secondly, there is a potential selection bias due to a differential loss to follow–up as there was no information in the Oracle database that recorded the continuous association of patients with the Oracle EHR Real-World Data network of medical facilities. Thirdly, there might be an uncounted placebo effect arising from the fact that patients on the combined treatment might expect to experience the largest possible benefit among drug treatments. Lastly, our study excluded 24.3% of the original sample due to the fact that these patients switched treatments at various time points after their initial diagnoses. In this group, a new analysis involving time-varying treatments will be necessary.

Therefore, further studies are needed to address these limitations and to better understand the potential benefits of combination therapy for Alzheimer’s disease patients.

## Conclusions

In this study, we implement a doubly robust causal inference approach to estimate the effect of no drug treatment, Memantine and Donepezil monotherapies, and Memantine and Donepezil combined treatment on the five-year survival of Alzheimer’s disease patients. To our knowledge, this is the first study utilizing causal inference with this objective and one of the largest and most comprehensive Alzheimer’s disease studies based on large sample size and high-quality data. This study reveals a significant, beneficial drug-drug interaction, indicating that the combined use of Memantine and Donepezil could significantly increase the probability of five-year survival in Alzheimer’s disease patients. Sensitivity analysis based on the e-value measure shows the robustness of our results to the impact of potential unmeasured confounders on both the estimated effect sizes and their corresponding confidence intervals. Our findings provide important evidence for the potential benefits of the combined therapy.

### Supplementary information

Reporting summary

## Data Availability

This study used a commercial database made available under license that the authors do not have permission to share. Request for access to the data should be directed to Oracle EHR Real-World Data at https://www.oracle.com/health/population-health/real-world-data/. We can provide our SQL, Python and R code for the queries and data cleaning upon request and with Oracle EHR Real-World Data approval.

## Code availability

The data analysis code used to produce the results presented in this paper is publicly available on GitHub at https://github.com/rakovski22/Causal_AD^31^.
